# Exploring conditions for implementing daily monitoring of Behavioral and Psychological Symptoms of Dementia (BPSD) in dementia care: insights from a participatory design study

**DOI:** 10.1186/s12877-026-07321-0

**Published:** 2026-03-11

**Authors:** Johannes Malm, Elzana Odzakovic, Ingemar Kåreholt, Sofi Fristedt, Therése Bielsten

**Affiliations:** 1 Institute of Gerontology, School of Health and Welfare, Jönköping University, Jönköping, Sweden; 2 Department of Nursing, School of Health and Welfare, Jönköping University, Jönköping, Sweden

**Keywords:** Assessment, BPSD, Dementia, Digital health, Implementation, User involvement, Person-centered care

## Abstract

**Background:**

Behavioral and Psychological Symptoms of Dementia (BPSD) affect up to 90% of people living with dementia and present significant challenges for individuals, relatives, and care staff, often impacting quality of life and care provision. Despite the high prevalence and fluctuating nature, BPSD are often monitored infrequently in routine care, limiting opportunities for timely, person-centered interventions. Digital tools that support frequent and individualized symptom monitoring may help bridge this gap.

**Methods:**

This study explored contextual factors, including barriers, facilitators, needs, and expectations, relevant to the potential implementation of Daily-BPSD, a digital tool designed to support individualized and frequent monitoring of BPSD in Swedish nursing homes. This participatory design study was conducted in two stages. Preparatory semi-structured interviews with disability care staff guided the initial design of the Daily-BPSD prototype, followed by research circles with nursing home staff and relatives. Data were analyzed thematically.

**Results:**

The analysis generated six themes: (1) implementation and adoption, (2) usability and accessibility, (3) communication and collaboration, (4) value for care and people with dementia, (5) training and support, and (6) the selection and interpretation of relevant variables to monitor. The findings were interpreted in relation to established determinants of technology acceptance, providing a structured understanding of factors shaping the anticipated adoption of Daily-BPSD.

**Conclusion:**

Findings emphasize the need to align digital tools with everyday work routines, ensure adequate training, and address infrastructural conditions for reliable use. By outlining conditions that may enable or hinder daily monitoring of BPSD, this study contributes to the development phase of complex intervention design. It provides a foundation for further refinement and feasibility testing of Daily-BPSD in dementia care.

**Supplementary Information:**

The online version contains supplementary material available at 10.1186/s12877-026-07321-0.

## Background

 People living with dementia experience a gradual decline in cognitive abilities, usually resulting in memory loss, difficulties with communication, impaired orientation and learning, and an inability to perform previously familiar tasks [1–4]. Globally, demographic shifts are marked by an increasing number and proportion of older adults, driven by population aging and higher life expectancy, leading to a projected rise in dementia cases worldwide from 60 million today to approximately 150 million by 2050 [5]. The rising prevalence of dementia will lead to an urgent need for public health interventions and tailored policies for people with dementia and their informal and formal caregivers [6–8].

Beyond cognitive decline, dementia also brings significant psychological and emotional effects. Feelings of frustration, inadequacy, and loss of independence are common, as the progressive nature of the condition challenges a person’s sense of identity and continuity of self [1, 9–11]. At some stage of the condition, as many as 90% of people living with dementia develop Behavioral and Psychological Symptoms of Dementia (BPSD) [2, 12]. These neuropsychiatric symptoms may include depression, apathy, delusions, motor disturbances, sleep disorders, and changes in appetite, and they often present differently across individuals [2, 12, 13]. BPSD can have a fundamental negative impact on quality of life and lead to considerable challenges for both those affected and their caregivers [14, 15].

Pharmacological interventions are commonly used but often show limited efficacy and may be associated with significant side effects. This has contributed to a growing consensus that non-pharmacological interventions should be the first-line approach in managing BPSD, emphasizing individualized, person-centered strategies tailored to the person’s needs and context [16–19]. Non-pharmacological, person-centered strategies such as cognitive stimulation, physical activity, music therapy, social interaction adaptations, and environmental adjustments have shown positive outcomes in reducing BPSD and improving emotional well-being [15, 20–22].

Person-centered care (PCC) is widely recognized as a guiding approach in dementia care, emphasizing the importance of understanding individuals’ needs, preferences, and everyday experiences within their care context [23]. Frameworks of PCC, such as those described by McCormack and McCance [24], highlight relational processes, shared decision-making, and supportive care environments as central to high-quality care. From this perspective, systematic and timely assessment of BPSD can support more responsive and coordinated care practices [25, 26]. Accurate identification and interpretation of symptoms are important prerequisites for enabling proactive, non-pharmacological interventions that align with PCC principles. Such practices require effective collaboration across professional roles and care settings [23, 27]. In addition, previous research has shown the importance of accurate assessment, identification, and interpretation of BPSD to enable timely interventions. Preventive and timely actions have demonstrated particular significance in reducing and mitigating adverse outcomes for people living with dementia and ensuring practices align with PCC [16, 23, 28, 29]. To meet the PCC standard, formal caregivers, such as nurses, physicians, support staff, and administrators, need to collaborate more effectively as a unified team. This requires not only accurate identification and interpretation of BPSD but also the ability to implement proactive non-pharmacological interventions [30, 31].

In this context, digital tools for structured and frequent symptom monitoring may support PCC by making individual patterns, fluctuations, and contextual triggers more visible in everyday practice. By supporting shared reflection and coordinated responses, such tools have the potential to strengthen continuity, personalization, and responsiveness in dementia care.

In Sweden, the BPSD Registry [32] has become a commonly used approach for systematically monitoring and managing symptoms of BPSD in individuals residing in nursing homes and receiving clinical care [33, 34]. Using the Neuropsychiatric Inventory (NPI) enables documentation of symptom occurrence, severity, and progression to inform evidence-based, individualized interventions [13, 35]. However, the routine in the BPSD registry relies on registrations only conducted every 3–12 months, which may delay the detection of both positive and negative changes and the evaluation of interventions.

Although most people living with dementia experience BPSD, and these symptoms are highly variable and can often lead to negative outcomes, nursing homes still lack solutions that allow for frequent monitoring and quick responses as the condition progresses. This highlights the importance of developing digital tools for more regular monitoring to enhance person-centered dementia care in nursing homes.

## Aim

This study aimed to identify contextual factors relevant to the implementation of frequent BPSD monitoring in Swedish nursing homes, including anticipated barriers and facilitators. These insights were used to inform the co-development of Daily-BPSD, a digital tool designed to support individualized and responsive care for people living with dementia.

## Methods

### Overall design

This qualitative participatory design study consisted of preparatory work and a main study. The preparatory work consisted of semi-structured interviews with disability care staff to explore experiences of a comparable digital follow-up tool. Insights from these interviews informed the design of the main study, which used research circles [36] with nursing home staff and relatives to identify contextual barriers and facilitators relevant to the future implementation of Daily-BPSD.

To structure the development process, the study was guided by the framework for developing and evaluating complex interventions by Skivington et al. [37] (see Table 1), situating the present work within the development phase. In line with Phase 1, we adapted a previously developed tool from disability care to the dementia care context, involving stakeholders to ensure contextual relevance and shared development.

**Table 1:** Phases in Skivington et al.’s framework for developing and evaluating complex interventions

Skivington’s Framework Phases		Project Activities
1.	Development or identification of intervention*	Preparatory work including interviews in disability care, drawing on experiences from an existing digital follow-up tool. The development of Daily-BPSD informed by research circles involving nursing home staff and relatives.
2.	Feasibility assessment	Pilot study to test Daily-BPSD in a care environment and examine usability and integration.
3.	Evaluation	Analyze pilot results, assess effects, and adjust the intervention based on caregivers’ experiences.
4.	Implementation	Implement Daily-BPSD on a larger scale and evaluate its long-term impact on care and work routines.

*The present study is limited to this phase of the framework

For theoretical interpretation, the Unified Theory of Acceptance and Use of Technology (UTAUT) [38] was applied post hoc to assess the alignment between empirically derived themes and established determinants of technology acceptance.

### Preparatory work: interviews with staff in disability care

The preparatory work aimed to explore how a previously developed digital tool for personalized, systematic follow-up had been implemented and experienced by staff. The tool had been used in disability care, where structured routines for daily monitoring were already established, making it a relevant reference for monitoring BPSD. The interviews were intended to identify challenges and facilitators that could inform the adaptation of a similar approach in dementia care.

### Participants and data collection

Participants were recruited from four municipalities in Sweden and included staff members working in group homes for individuals with disabilities, all with experience using the previously developed digital follow-up tool. The sample included personal assistants (*n* = 7), care unit managers (*n* = 3), and one activity coordinator. All participants provided verbal informed consent prior to participation. Data collection involved seven semi-structured individual interviews and one group interview (*n* = 4), conducted via video conferencing. Interviews were led by researchers Sofi Fristedt (SF), associate professor in health and caring sciences and a council-certified specialist in occupational therapy, and/or Therése Bielsten (TB), senior lecturer in gerontology and a specialist nurse in dementia care, and lasted between 12 and 71 min. All were audio-recorded and transcribed verbatim.

### Data analysis

Interview data were analyzed using an interpretative qualitative approach with an inductive thematic analysis. The analysis was informed by principles of constant comparison [37], where codes and emerging patterns were continuously compared across interviews to explore similarities and variations. Transcripts were read repeatedly, and open coding was conducted to identify recurring concepts. Codes were iteratively compared across interviews to explore similarities and variations, leading to the refinement of categories and development of overarching themes. Ongoing discussions within the research team supported interpretation and helped ensure the analytical categories were grounded in the data

### Key insights

Four key areas emerged from the analysis:

- Implementation and Training: Successful adoption was supported by stepwise introduction, clearly assigned responsibilities, and ongoing training. The presence of internal “superusers” helped maintain engagement. Initial skepticism often shifted as staff became more familiar with the system.
- Integration and Digital Access: While the tool was described as time-efficient, barriers related to infrastructure and access to devices for entering data, especially during night shifts, limited consistent use. Staff were more motivated when they could see how data-informed care decisions were made.
- Variable Selection and Reliability: Participants reported uncertainty about which variables (individualized aspects of the person’s health, behavior, or well-being) should be included and how to interpret them consistently. Teams often adjusted variables over time and established internal guidelines to facilitate consistent interpretation of scales. Despite these efforts, inconsistencies persisted in how variables were understood and applied, particularly when temporary staff, who may not have received the same training, were involved.
- Data Use in Practice: When monitoring reports were used in team meetings, staff reported improvements in care planning. In contrast, inconsistent data entry undermined the reports’ usefulness and decreased overall trust in and engagement with the system.

### Main study

Insights from the preparatory work served as a foundation and guided the planning and execution of the research circles. An initial prototype of Daily-BPSD was developed, prior to the main study data collection, to provide participants with a reference point.

### Design

The main study applied an exploratory qualitative design through research circles. Research circles, as described by Härnsten [36] and further developed by Iwarsson et al. [39] and Löfqvist et al. [40] is a participatory and dialogical methodology grounded in principles of knowledge co-production. In this approach, researchers and practitioners meet in recurring group discussions to jointly discuss problems and generate knowledge on more equal terms, ensuring that research is both contextually relevant and grounded in practice.

A prototype of Daily-BPSD was presented during the initial research circle to support discussions of needs, expectations, and the tool’s perceived strengths and limitations. These discussions informed reflections and considerations relevant to the continued development of Daily-BPSD beyond the study. The research circles functioned as an exploratory qualitative approach to inform design decisions and did not aim to carry out a fully iterative co-design process involving repeated prototyping and re-validation.

### Participants

Participants were recruited with assistance from unit care managers from five nursing homes located in two municipalities in southern Sweden. Participants were assigned to three parallel research circles, with two groups composed of staff from multiple facilities and one group from a single site. The recruitment aimed to include a variety of professional roles and experiences, in line with recommendations for heterogeneity to enhance practical relevance [40]. All participants gave verbally informed consent prior to participation.

### Data collection

Each research circle met twice and followed a structured guide (see Table 2), developed by the research team based on themes from the preparatory work. In all groups, the first session began with problem orientation through a summary of the preparatory work and a demonstration of the developed prototype of Daily-BPSD. Sessions were held face-to-face at the participating facilities and were audio-recorded. This aimed to establish similar starting points and a common frame of reference. Participants were encouraged to move the discussions forward with guidance from the researchers, whose insights were primarily based on the preparatory work and the prototype’s potential strengths and limitations. In line with the research circle methodology, researchers participated in the dialogue as knowledge contributors rather than solely as neutral moderators. At the same time, attention was paid to minimizing directional bias; researchers avoided evaluative positioning, actively invited divergent viewpoints, and emphasized that there were no predetermined “correct” solutions. Following each session, the research team conducted reflexive discussions to critically consider how the researcher’s assumptions or framing might have influenced the dialogue (Table 2).

**Table 2:** Summary of research circles questioning and theming guides. Full guides are available in the appendix

**Meeting I – Questions**	
1. Can you tell us about your experience working with people with BPSD? 2. What do you consider to be particularly important to follow up on a daily basis for people with BPSD? 3. What kind of assessments do you conduct today, in addition to registrations in the BPSD registry? 4. Can you describe how results from assessments are used today? 5. What do you consider important that we continue to discuss further at meeting II?	
**Meeting II – Topics**	
1. The BPSD registry 2. Assessments in nursing homes 3. Variable selection: what is relevant to follow-up? 4. Routines and responsibilities 5. Use of results 6. Potential value and challenges	

At the end of the first session, participants across the groups were asked what they considered important to discuss during session two—to address the problem as comprehensively as possible. The first session focused primarily on current practices related to BPSD assessment and follow-up. Participants shared their experiences, challenges, and routines concerning symptom monitoring. Between sessions, data from session one was organized, analyzed, and discussed by the research team. Insights from this process helped shape the focus of session two. In the second session, discussions built on themes from the first and addressed how relevant variables for the tool could be selected, who should take responsibility for data entry and follow-up, and how documentation routines could be organized. The groups also discussed the expected benefits and challenges of implementing a new digital tool before any practical testing in care settings.

Interviews were conducted in Swedish and translated into English using AI-assisted translation tools. All translations were subsequently reviewed by the authors to ensure preservation of meaning and contextual nuance. Quotations were minimally edited for readability while retaining their original intent.

### Analysis

Data were analyzed using inductive thematic analysis [41]. Transcripts were read repeatedly to achieve familiarity with the data. An inductive, data-driven coding process was then applied to identify relevant patterns across the material. Coded extracts were organized into preliminary themes, which were iteratively reviewed, refined, and discussed within the research team to ensure internal coherence and alignment with the study aim. Final themes were defined to capture core meanings regarding anticipated barriers and facilitators to implementing daily monitoring of BPSD. Coding and theme development were carried out collaboratively, with regular peer debriefing sessions to support reflexivity and analytical rigor.

Given the participatory and practice-based nature of the research circles, the analytic strategy was designed to capture experiential knowledge from both formal caregivers (e.g. nursing staff, managers, and allied professionals) and informal caregivers (relatives), while also informing the development of a digital support tool for dementia care. The inductive approach was considered particularly appropriate given the tacit, relational, and context-dependent nature of dementia care, as well as its alignment with participatory methodologies that emphasize lived experience and the co-creation of knowledge [42, 43].

To support theoretical interpretation of the findings, the final themes were reflected upon in relation to established theories of technology adoption in healthcare. UTAUT [44] was used as an interpretative lens to contextualize the identified barriers and facilitators. UTAUT was not used in the coding process or theme development but was applied post hoc to support analytical interpretation and to inform an interpretative synthesis of the inductively derived themes, situating the findings within broader implementation and technology-acceptance literature.

Accordingly, the analytic approach was primarily inductive and thematic, with abductive reasoning applied at the interpretative stage to relate empirically grounded findings to existing theory.

## Results

In total, 31 individuals took part: 12 assistant nurses covering both day and night shifts, four first-line care managers, five registered nurses, four members of dementia support teams, three researchers (JM, TB, IK), two relatives, and one occupational therapist. The findings were categorized into six themes: *implementation and adoption*, *usability and accessibility*, *communication and collaboration*, *value for care and people with dementia*, *training and ongoing support*, and *variable selection*.

### Implementation and adoption

Introducing new digital systems in care settings is often expected to disrupt established routines and require both behavioral and organizational adaptations. This theme reflects the anticipated structural, practical, and psychological barriers that participants believed could hinder the adoption of systems like Daily-BPSD, including uncertainty, resistance to change, and lack of support structures.

Participants expressed concerns about how such a system would be integrated into existing routines, particularly in the absence of clear guidance and sufficient training. These perceived gaps were thought to potentially lead to hesitation and inconsistent use. One participant remarked: *“I find it difficult with this duplication*,* because what we document in the medical record we must continue with.”* (Participant 9, Unit Manager). Such reflections highlight the importance of coherent implementation strategies that minimize redundant documentation across different systems and ensure clarity in documentation processes.

### Usability and accessibility

Usability refers to how easy and efficient a digital application is to use, while accessibility concerns whether users can access and operate it regardless of technical or contextual barriers. Participants emphasized that both aspects would be critical for the successful integration of Daily-BPSD into everyday care routines.

A recurring concern was that digital systems might be difficult to navigate or poorly aligned with the workflows of care staff. Participants anticipated that unfamiliar or complex interfaces could become time-consuming and stressful in an already demanding work environment. This highlighted the perceived need for user-friendly design and readily accessible functions.

One participant expressed concern about how information would be delivered and interpreted, stating: *“Even if it’s important information*,* I’m not sure how it actually can be used in practice.”* (Participant 25, Researcher). Such reflections underscore the importance of not only providing relevant data but also doing so in a way that feels manageable and meaningful to staff in their daily work.

### Communication and collaboration

In the context of dementia care, communication refers to the flow of information between different professional roles, while collaboration involves coordinated efforts to ensure continuity and quality in care. Participants emphasized that both are essential for the successful introduction of new systems, such as Daily-BPSD.

Several participants anticipated that unclear communication channels or limited cross-professional collaboration could hinder effective use of the tool. They expressed concern that insufficient access to shared information might create confusion, particularly when different staff categories use different documentation systems.

One participant noted: *“We*,* as nurses and rehab staff*,* don’t have access to certain information.”* (Participant 13, Nurse). These reflections illustrate how structural and organizational gaps in information sharing could undermine the intended benefits of a digital monitoring system—unless explicitly addressed during implementation planning.

### Value for care and people with dementia

In this context, value refers to the anticipated benefits a tool like Daily-BPSD could bring to both care quality and the everyday experience of people living with dementia. Participants reflected on how digital monitoring might support more timely, individualized care, assuming the tool is meaningfully integrated into daily routines.

Some participants expected that such a tool could increase awareness of behavioral changes and improve continuity in care planning. They also believed that systematic monitoring might offer a clearer picture of residents’ day-to-day needs, enabling more proactive interventions.

As one participant suggested, *“More knowledge captures everyone’s attention. It’s not just about care but also about nurturing. It can actually broaden one’s perspective on their job*,* which is interesting.”* (Participant 19, Assistant Nurse). These expectations highlight the perceived potential of Daily-BPSD to strengthen person-centered care, not only by improving documentation but also by deepening staff insight into residents’ behavioral expressions and well-being.

### Training and ongoing support

Participants emphasized the importance of both initial training and ongoing support to enable confident and consistent use of a future system, such as Daily-BPSD. They highlighted the need for clear guidelines, opportunities for continued learning, and reliable technical assistance. These elements were considered essential for ensuring that staff could understand and effectively apply the tool in a meaningful way.

The need for accessible and consistent routines was particularly emphasized with regard to temporary or rotating staff, whose limited onboarding may result in less reliable documentation. One participant noted, *“Now*,* I think apathy [one of the symptoms of BPSD] is a matter of interpretation; what do you mean when you say apathy?”* (Participant 2, Assistant Nurse). This underlines the importance of a shared understanding of key concepts.

Beyond initial instruction, participants also called for structures that support sustained use over time. As one participant put it: *“Even though there is a shared responsibility*,* as you have*,* it may still be necessary to set specific times for tasks to be completed. Regardless of who performs the task*,* it is a routine that needs to be established.”* (Participant 7, Assistant Nurse). This reflects a perceived need for clear routines, reminders, and reinforcement to support lasting integration into daily practice.

### Variable selection

Participants identified the choice of variables, specifically which aspects of residents’ behavior, health, or well-being to monitor, as a key concern in relation to future implementation. One of the main anticipated challenges was finding a balance between capturing sufficient information and keeping the process manageable in everyday workflows. Monitoring too many variables was seen as potentially overwhelming, while monitoring too few might not provide a meaningful picture of the resident’s needs.

Participants discussed concerns about the relevance, feasibility, and clarity of certain variables. All groups highlighted NPI variables as a relevant foundation for future documentation with Daily-BPSD but stressed the importance of flexibility and individual adaptation.

Variable selection reflects the tension between the desire to collect meaningful data that could support outcomes for people with dementia and the practical need to maintain usability. As one participant noted, *“It would become clear that for those who have these symptoms*,* it is indeed important to do the registration. But I also understand the time constraints. Therefore*,* I think it is especially important to do registrations for those who have early symptoms.”* (Participant 29, Relative). Another participant added: *“Shouldn’t you only fill in what is current and relevant?”* (Participant 12, Nurse). These reflections illustrate how staff and relatives balanced the perceived clinical value of comprehensive documentation against the realities of limited time and workload, thereby underscoring the need for tailored variables that remain both meaningful and manageable.

### Synthesis of findings in relation to UTAUT

To clarify how the inductively derived themes relate to established determinants of technology acceptance, Table 3 presents an interpretative mapping of the findings to the four core determinants specified in the UTAUT framework: performance expectancy, effort expectancy, social influence, and facilitating conditions.

**Table 3:** Interpretative mapping of inductively derived themes to UTAUT core determinants

Theme	UTUAT determinant	Interpretation
Implementation and adoption	Facilitating conditions	Organizational readiness, system integration, and infrastructural support
Usability and accessibility	Effort expectancy	Cognitive load, usability, accessibility, and time demands in daily work
Communication and collaboration	Social influence	Leadership engagement, shared routines, and cross-professional norms
Value for care and people with dementia	Performance expectancy	Anticipated benefits such as timely, individualized care and improved understanding of residents’ needs
Training and ongoing support	Facilitating conditions	Access to training, guidance, and ongoing technical support
Variable selection	(not interpreted as one of UTAUT’s determinants)	Balance between clinical relevance and manageable documentation

## Discussion

This study explored contextual barriers and facilitators relevant to the future implementation of Daily-BPSD, a digital tool for daily monitoring of BPSD in people with dementia living in nursing homes. By involving staff and relatives in research circles, the study identified key conditions that could either support or hinder adoption and integration into practice. Framing the work within a co-development approach means that the findings not only highlight barriers and facilitators but also offer practical guidance for refining Daily-BPSD in collaboration with stakeholders. In this way, the study contributes to the participatory design tradition, where knowledge is created jointly to ensure both contextual relevance and long-term usability [38, 45, 46].

The convergence between the inductively derived themes and established UTAUT determinants strengthens the interpretative validity of the findings. However, the theme of variable selection was less straightforward to align with UTAUT, as participants emphasized clinical relevance in addition to manageability and effort. This suggests that perceived content relevance may influence attitudes toward digital tools, an aspect that has been noted as less explicitly addressed in the UTAUT framework [47] and may still be critical for acceptance in dementia care.

One prominent factor shaping anticipated adoption is the concern regarding double documentation, which highlights a broader structural challenge related to digital fragmentation in long-term care. Although Daily-BPSD was perceived as adding clinical value, its adoption is constrained by limited interoperability among existing documentation systems, indicating a need for system-level solutions beyond local implementation efforts. Accordingly, the findings suggest that successful implementation depends not only on local usability and staff engagement but also on alignment with broader organizational and policy-level initiatives supporting interoperable digital care systems.

Given the sensitivity of resident-related behavioral data, implementation also requires clear definitions of how data are collected, who has role-based access, how information is used for clinical decision-making, and how long records are retained. Access should be carefully regulated according to professional roles, with clearly defined responsibilities and accountability to ensure appropriate use of information and maintain trust.

Daily monitoring was seen as potentially improving team communication, care planning, and responsiveness to behavioral changes, thereby adding value to the existing Swedish BPSD registry, which documents symptoms only periodically. This perspective resonates with earlier studies showing the benefits of frequent monitoring for timely detection and intervention [48–50].

However, realizing these benefits requires careful attention to governance and implementation conditions. Although designed to support learning and person-centered care, such tools may be perceived as instruments of staff surveillance if governance structures are unclear, potentially affecting the risk of technology rejection [51] and diminishing staff sense of agency and responsibility [52]. Longitudinal symptom records may also contribute to labeling if not interpreted contextually. Clear purpose limitation, transparent access rules, and a non-punitive learning orientation are therefore essential to ensure that Daily-BPSD supports care improvement rather than disciplinary use.

Participants pointed to several practical requirements that must be addressed before piloting Daily-BPSD in care settings:

- Documentation routines – Mapping current practices and clarifying how Daily-BPSD integrates with, rather than duplicates, existing systems.
- Training and onboarding – Providing clear guidelines, recurrent training, and routines for temporary or new staff to ensure consistent use.
- Technical feasibility – Securing reliable access to devices, stable internet, and responsive IT support across facilities.
- User friendliness – Refining the user interface to minimize the time required for documentation and to facilitate intuitive handling for staff, while also supporting meaningful interactions with people with dementia.
- Leadership engagement – Promoting the purpose of Daily-BPSD, fostering ownership among staff, and encouraging open dialogue during transition.

These insights highlight how technical, organizational, and cultural readiness intersect in shaping the adoption of digital tools, consistent with evidence that infrastructural capacity, user friendliness, and professional engagement and confidence are decisive for successful implementation [53–55]. The study did not identify the potential financial barriers to adoption, such as investment costs and economic incentives, which are cited in the literature as potential obstacles to implementation [55, 56]. By not addressing these financial considerations, the analysis may underestimate the structural challenges that organizations face when deciding whether to invest in and sustain a new digital tool.

Current documentation systems were perceived as insufficient for individualized monitoring and decision-making, which limits alignment with PCC. Daily-BPSD was seen as a potential complement that could capture daily fluctuations in symptoms and provide a richer basis for tailoring interventions. This reframes digital documentation not merely as an administrative burden, but as a practical resource for strengthening personhood, continuity, and responsiveness in dementia care.

### Strengths and limitations

The study was guided by principles of naturalistic inquiry, in which trustworthiness is assessed through credibility, transferability, dependability, and confirmability [57].

*Credibility* was strengthened by involving and gathering experiences from various professions within nursing home settings, relatives, and researchers. The two-session iteration aimed to enhance authenticity, but representativeness might have been limited by the uneven distribution of roles. The research team brought complementary expertise in gerontology, dementia care, and qualitative research, which supported informed data collection and interpretation and facilitated awareness of potential interpretative bias. Given the participatory research circle approach, researchers held a dual role as both contributors to the dialogue and analysts of the material. Reflexive discussions were therefore conducted throughout the process to critically examine how researcher positioning and disciplinary perspectives might have shaped the framing of issues or the interpretation of findings.

The hierarchical care context may have influenced group dynamics and participants’ willingness to express critical views. To mitigate this, the research circles emphasized open dialogue and reflexivity, actively encouraging contributions from all participants. Nevertheless, potential power relations should be acknowledged. The number of relatives was small (*n* = 2), but they still provided additional perspectives. Given the study’s focus on organizational and professional implementation conditions, staff perspectives predominated, and further research is needed to explore relatives’ experiences.

*Transferability* was supported by including various professional roles and by the fact that the nursing home care settings share many similarities across Sweden, although it may not be directly applicable to countries outside Sweden. The focus on shared variables limited the exploration of more individual symptoms, and a more thorough comparison with existing systems could have clarified the tool’s unique contribution. *Dependability* benefited from organized documentation of the iterative design process and multi-researcher analysis. The nature of the research circle approach is to allow discussions to evolve somewhat freely, which likely leads to decreased consistency across data. *Confirmability* was enhanced through team reflexivity and collaborative coding, though the lack of triangulation and reliance on anticipated rather than experienced tool use limited external verification. Enabling participants to interact with the prototype might have yielded more grounded insights.

### Future research directions

This study contributes to the development stage of the framework for complex interventions [58]. Future research will focus on the feasibility testing of Daily-BPSD in routine dementia care settings. This includes examining how the tool integrates into everyday workflows, its usability from the perspective of different staff roles, and the organizational conditions that support or hinder consistent use. Evaluation is intended to extend beyond adoption rates to include completion rates, documentation time, variation in staff engagement, integration into care planning, and whether registrations appear to trigger care actions. Staff perceptions of workload and value creation will also be explored to better understand how the tool could be embedded in practice.

Further research could also explore how Daily-BPSD complements existing documentation systems, such as the Swedish BPSD registry. Comparative studies may clarify whether the tool offers added value, supports clinical decision-making, or introduces redundancy. Beyond technical interoperability, future system-level solutions may also benefit from data-driven approaches that enable information from multiple documentation systems to be interpreted and synthesized rather than redundantly recorded. For example, analytic methods such as natural language processing could support the integration of unstructured clinical notes across systems, thereby enhancing informational value while reducing documentation burden.

As the tool evolves, it will be important to consider how flexibility can be balanced with standardization. While shared variables are essential for comparability, the ability to tailor content to individual needs, such as specific symptoms, behaviors, daily activities, or care actions, may enhance the tool’s clinical relevance. Exploring approaches like adaptive variable templates or case-based extensions could support a more person-centered and responsive use of the tool.

In later stages, outcome evaluations should investigate whether the tool contributes to improved symptom recognition, more proactive care planning, and strengthened person-centered practices in dementia care.

## Conclusion

This study identified key contextual barriers and facilitators for the future implementation of Daily-BPSD, a digital tool designed to support daily monitoring of behavioral and psychological symptoms in dementia care. While not yet tested in practice, the findings contribute to the development phase of complex intervention design by outlining conditions that may enable or hinder successful integration in nursing home settings.

The findings highlight the importance of aligning digital tools with existing routines, ensuring adequate staff training, and addressing infrastructural limitations. The possibility to adapt documentation content to individual needs—such as symptoms, behaviors, care actions, or daily activities—emerged as particularly important for usability and relevance in everyday practice. Daily-BPSD has the potential to complement existing assessments, such as the Swedish BPSD registry, by capturing daily fluctuations and enabling more responsive and person-centered care planning.

The participatory approach, involving care staff and relatives through research circles, aimed to strengthen the contextual relevance and usability of the tool. By grounding design considerations in lived experience and practical knowledge, the study offers insights into how digital documentation tools can be meaningfully adapted to the realities of dementia care.

These findings lay the groundwork for ongoing co-development, feasibility testing, and refinement—supporting future work that will evaluate the tool in practice, compare it with existing systems, and fine-tune the balance between personalization and standardized documentation.

## Supplementary Information

Supplementary Material 1.

## Data Availability

The data collected and analyzed during the current study are not publicly available. The qualitative material contains sensitive information regarding participating nursing homes, staff, and relatives. To protect the identity of the care providers and individual participants, the data cannot be made publicly accessible.

## Abbreviations

BPSD: Behavioral and Psychological Symptoms of Dementia
NPI: Neuropsychiatric Inventory
PCC: Person-Centered Care
UTAUT: Unified Theory of Acceptance and Use of Technology

## References

[1] Bielsten T. Doing things together: towards a health promoting approach to couples’ relationships and everyday life in dementia. Linköping: Linköping University Electronic; 2020. 10.3384/diss.diva-164273.

[2] Cerejeira J, Lagarto L, Mukaetova-Ladinska EB. Behavioral and psychological symptoms of dementia. Front Neurol. 2012;3. 10.3389/fneur.2012.00073. 10.3389/fneur.2012.00073 PMC3345875 22586419

[3] Duong S, Patel T, Chang F. Dementia: what pharmacists need to know. Can Pharm J. 2017;150:118–29. 10.1177/1715163517690745. 10.1177/1715163517690745 PMC5384525 28405256

[4] Savva GM, Zaccai J, Matthews FE, Davidson JE, McKeith I, Brayne C. Prevalence, correlates and course of behavioural and psychological symptoms of dementia in the population. Br J Psychiatry. 2009;194:212–9. 10.1192/bjp.bp.108.049619. 19252147 10.1192/bjp.bp.108.049619

[5] GBD 2019 Dementia Forecasting Collaborators. Estimation of the global prevalence of dementia in 2019 and forecasted prevalence in 2050: an analysis for the Global Burden of Disease Study 2019. Lancet Public Health. 2022;7:e105–25. 10.1016/S2468-2667(21)00249-8. 34998485 10.1016/S2468-2667(21)00249-8 PMC8810394

[6] Alzheimer’s Disease International. Dementia statistics. 2024. https://www.alzint.org/about/dementia-facts-figures/dementia-statistics/. Accessed 9 Sept 2025.

[7] Rangarajan SK, Sivakumar PT, Manjunatha N, Kumar CN, Math SB. Public health perspectives of geriatric mental health care. Indian J Psychol Med. 2021;43 5suppl:S1–7. 10.1177/02537176211047963. 34732947 10.1177/02537176211047963 PMC8543608

[8] World Health Organization. Dementia, fact sheet. 2025. https://www.who.int/news-room/fact-sheets/detail/dementia. Accessed 4 Aug 2025.

[9] Beard RL. In their voices: Identity preservation and experiences of Alzheimer’s disease. J Aging Stud. 2004;18:415–28. 10.1016/j.jaging.2004.06.005.

[10] Górska S, Forsyth K, Maciver D. Living with dementia: a meta-synthesis of qualitative research on the lived experience. Gerontologist. 2018;58:e180–96. 10.1093/geront/gnw195. 28069886 10.1093/geront/gnw195 PMC5946830

[11] Kitwood TM. Dementia reconsidered: the person comes first. Buckingham: Open University, Buckingham, UK;; 1997.

[12] Laganà V, Bruno F, Altomari N, Bruni G, Smirne N, Curcio S, et al. Neuropsychiatric or behavioral and psychological symptoms of dementia (BPSD): focus on prevalence and natural history in Alzheimer’s disease and frontotemporal dementia. Front Neurol. 2022;13:832199. 10.3389/fneur.2022.832199. 35812082 10.3389/fneur.2022.832199 PMC9263122

[13] Cummings JL, Mega M, Gray K, Rosenberg-Thompson S, Carusi DA, Gornbein J. The neuropsychiatric inventory: comprehensive assessment of psychopathology in dementia. Neurology. 1994;44:2308–14. 10.1212/wnl.44.12.2308. 7991117 10.1212/wnl.44.12.2308

[14] Mukherjee A, Biswas A, Roy A, Biswas S, Gangopadhyay G, Das SK. Behavioural and psychological symptoms of dementia: correlates and impact on caregiver distress. Dement Geriatr Cogn Disord Extra. 2017;7:354–65. 10.1159/000481568. 10.1159/000481568 PMC5731149 29282408

[15] Testad I, Corbett A, Aarsland D, Lexow KO, Fossey J, Woods B, et al. The value of personalized psychosocial interventions to address behavioral and psychological symptoms in people with dementia living in care home settings: a systematic review. Int Psychogeriatr. 2014;26:1083–98. 10.1017/S1041610214000131. 24565226 10.1017/S1041610214000131

[16] Bessey LJ, Walaszek A. Management of behavioral and psychological symptoms of dementia. Curr Psychiatry Rep. 2019;21:66. 10.1007/s11920-019-1049-5. 31264056 10.1007/s11920-019-1049-5

[17] Calsolaro V, Femminella GD, Rogani S, Esposito S, Franchi R, Okoye C, et al. Behavioral and psychological symptoms in dementia (BPSD) and the use of antipsychotics. Pharmaceuticals. 2021;14:246. 10.3390/ph14030246. 33803277 10.3390/ph14030246 PMC8002184

[18] Kales HC, Lyketsos CG, Miller EM, Ballard C. Management of behavioral and psychological symptoms in people with Alzheimer’s disease: an international Delphi consensus. Int Psychogeriatr. 2019;31:83–90. 10.1017/S1041610218000534. 30068400 10.1017/S1041610218000534

[19] Meng X, Su J, Li H, Ma D, Zhao Y, Li Y, et al. Effectiveness of caregiver non-pharmacological interventions for behavioural and psychological symptoms of dementia: an updated meta-analysis. Ageing Res Rev. 2021;71:101448. 10.1016/j.arr.2021.101448. 34416379 10.1016/j.arr.2021.101448

[20] Ihara ES, Tompkins CJ, Inoue M, Sonneman S. Results from a person-centered music intervention for individuals living with dementia. Geriatr Gerontol Int. 2019;19:30–4. 10.1111/ggi.13563. 30460747 10.1111/ggi.13563

[21] Mitchell G, Agnelli J. Person-centred care for people with dementia: Kitwood reconsidered. Nurs Stand. 2015;30:46–50. 10.7748/ns.30.7.46.s47. 26463810 10.7748/ns.30.7.46.s47

[22] Oliveira AMD, Radanovic M, Mello PCHD, Buchain PC, Vizzotto ADB, Celestino DL, et al. Nonpharmacological interventions to reduce behavioral and psychological symptoms of dementia: a systematic review. BioMed Res Int. 2015;2015:1–9. 10.1155/2015/218980. 10.1155/2015/218980 PMC4676992 26693477

[23] Lee KH, Lee JY, Kim B. Person-centered care in persons living with dementia: a systematic review and meta-analysis. Gerontologist. 2022;62:e253–64. 10.1093/geront/gnaa207. 33326573 10.1093/geront/gnaa207 PMC9019632

[24] McCormack B, McCance TV. Development of a framework for person-centred nursing. J Adv Nurs. 2006;56:472–9. 10.1111/j.1365-2648.2006.04042.x. 17078823 10.1111/j.1365-2648.2006.04042.x

[25] Kales HC, Gitlin LN, Lyketsos CG. Assessment and management of behavioral and psychological symptoms of dementia. BMJ. 2015;350:h369. 10.1136/bmj.h369. 10.1136/bmj.h369 PMC4707529 25731881

[26] Kolanowski A, Boltz M, Galik E, Gitlin LN, Kales HC, Resnick B, et al. Determinants of behavioral and psychological symptoms of dementia: A scoping review of the evidence. Nurs Outlook. 2017;65:515–29. 10.1016/j.outlook.2017.06.006. 28826872 10.1016/j.outlook.2017.06.006 PMC6579119

[27] Ekman I, Swedberg K, Taft C, Lindseth A, Norberg A, Brink E, et al. Person-centered care — ready for prime time. Eur J Cardiovasc Nurs. 2011;10:248–51. 10.1016/j.ejcnurse.2011.06.008. 21764386 10.1016/j.ejcnurse.2011.06.008

[28] Sefcik JS, Madrigal C, Heid AR, Molony SL, Van Haitsma K, Best I, et al. Person-centered care plans for nursing home residents with behavioral and psychological symptoms of dementia. J Gerontol Nurs. 2020;46:17–27. 10.3928/00989134-20201012-03. 33095889 10.3928/00989134-20201012-03 PMC8274316

[29] Van Der Linde RM, Dening T, Stephan BCM, Prina AM, Evans E, Brayne C. Longitudinal course of behavioural and psychological symptoms of dementia: systematic review. Br J Psychiatry. 2016;209:366–77. 10.1192/bjp.bp.114.148403. 27491532 10.1192/bjp.bp.114.148403 PMC5100633

[30] Fazio S, Zimmerman S, Doyle PJ, Shubeck E, Carpenter M, Coram P, et al. What is really needed to provide effective, person-centered care for behavioral expressions of dementia? Guidance from the Alzheimer’s Association dementia care provider roundtable. J Am Med Dir Assoc. 2020;21:1582–e15861. 10.1016/j.jamda.2020.05.017. 32723533 10.1016/j.jamda.2020.05.017

[31] Isaac V, Kuot A, Hamiduzzaman M, Strivens E, Greenhill J. The outcomes of a person-centered, non-pharmacological intervention in reducing agitation in residents with dementia in Australian rural nursing homes. BMC Geriatr. 2021;21:193. 10.1186/s12877-021-02151-8. 33743597 10.1186/s12877-021-02151-8 PMC7980426

[32] The BPSD, Registry. http://www.bpsd.se. Accessed 2 June 2025.

[33] Jönsson L, Wibom M, Londos E, Nägga K. Person-centered care at population scale: the Swedish registry for behavioral and psychological symptoms of dementia. Alzheimers Dement Transl Res Clin Interv. 2025;11:e70057. 10.1002/trc2.70057. 10.1002/trc2.70057 PMC11848623 39995597

[34] Schwertner E, Pereira JB, Xu H, Secnik J, Winblad B, Eriksdotter M, et al. Behavioral and psychological symptoms of dementia in different dementia disorders: A large-scale study of 10,000 individuals. J Alzheimers Dis. 2022;87:1307–18. 10.3233/JAD-215198. 35491774 10.3233/JAD-215198 PMC9198804

[35] The National Board of Health and Welfare. National Guidelines – Assessment 2018: Care and Support for People with Dementia – Summary and Areas for Improvement. Stockholm: Socialstyrelsen; 2018.

[36] Härnsten G. The research circle–building knowledge on equal terms. Stockholm: Swedish Trade Union Confederation (LO); 1994.

[37] Glaser BG. The constant comparative method of qualitative analysis. Soc Probl. 1965;12:436–45. 10.2307/798843.

[38] Bird M, McGillion M, Chambers EM, Dix J, Fajardo CJ, Gilmour M, et al. A generative co-design framework for healthcare innovation: development and application of an end-user engagement framework. Res Involv Engagem. 2021;7:12. 10.1186/s40900-021-00252-7. 33648588 10.1186/s40900-021-00252-7 PMC7923456

[39] Iwarsson S, Edberg A-K, Ivanoff SD, Hanson E, Jönson H, Schmidt S. Understanding user involvement in research in aging and health. Gerontol Geriatr Med. 2019;5:2333721419897781. 10.1177/2333721419897781. 31909093 10.1177/2333721419897781 PMC6937534

[40] Löfqvist C, Månsson Lexell E, Nilsson MH, Iwarsson S. Exploration of the research circle methodology for user involvement in research on home and health dynamics in old age. J Hous Elder. 2019;33:85–102. 10.1080/02763893.2018.1534176.

[41] Braun V, Clarke V. Conceptual and design thinking for thematic analysis. Qual Psychol. 2022;9:3–26. 10.1037/qup0000196.

[42] Azungah T. Qualitative research: deductive and inductive approaches to data analysis. Qual Res J. 2018;18:383–400. 10.1108/QRJ-D-18-00035.

[43] Greenhalgh T, Jackson C, Shaw S, Janamian T. Achieving Research Impact Through Co-creation in Community-Based Health Services: Literature Review and Case Study. Milbank Q. 2016;94:392–429. 10.1111/1468-0009.12197. 27265562 10.1111/1468-0009.12197 PMC4911728

[44] Venkatesh V, Morris MG, Davis GB, Davis FD. User Acceptance of Information Technology: Toward a Unified View. MIS Q. 2003;27:425–78. 10.2307/30036540.

[45] Bødker S, Dindler C, Iversen OS, Smith RC. What is participatory design? Participatory design. Springer; 2022. pp. 5–13.

[46] Spinuzzi C. The methodology of participatory design. Tech Commun. 2005;52:163–74.

[47] Dwivedi YK, Rana NP, Jeyaraj A, Clement M, Williams MD. Re-examining the Unified Theory of Acceptance and Use of Technology (UTAUT): Towards a Revised Theoretical Model. Inf Syst Front. 2019;21:719–34. 10.1007/s10796-017-9774-y.

[48] Dubois B, Padovani A, Scheltens P, Rossi A, Dell’Agnello G. Timely diagnosis for alzheimer’s disease: a literature review on benefits and challenges. J Alzheimers Dis. 2015;49:617–31. 10.3233/JAD-150692. 10.3233/JAD-150692 PMC4927869 26484931

[49] Husebo BS, Heintz HL, Berge LI, Owoyemi P, Rahman AT, Vahia IV. Sensing technology to monitor behavioral and psychological symptoms and to assess treatment response in people with dementia. A systematic review. Front Pharmacol. 2020;10:1699. 10.3389/fphar.2019.01699. 32116687 10.3389/fphar.2019.01699 PMC7011129

[50] Moyle W. The promise of technology in the future of dementia care. Nat Rev Neurol. 2019;15:353–9. 10.1038/s41582-019-0188-y. 31073242 10.1038/s41582-019-0188-y

[51] Abraham M, Niessen C, Schnabel C, Lorek K, Grimm V, Möslein KM, et al. Electronic monitoring at work: The role of attitudes, functions, and perceived control for the acceptance of tracking technologies. Hum Resour Manag J. 2019. 10.1111/1748-8583.12250.

[52] Thiel CE, Bonner JM, Bush J, Welsh DT, Garud N. Stripped of Agency: The Paradoxical Effect of Employee Monitoring on Deviance. J Manag. 2021;49:709–40. 10.1177/01492063211053224.

[53] AlQudah AA, Al-Emran M, Shaalan K. Technology acceptance in healthcare: a systematic review. Appl Sci. 2021;11:10537. 10.3390/app112210537.

[54] Burmann A, Schepers S, Meister S. Acceptance factors of digitalization in hospitals: a mixed-methods study. Health Technol. 2023;13:843–59. 10.1007/s12553-023-00779-7.

[55] Gagnon M-P, Desmartis M, Labrecque M, Car J, Pagliari C, Pluye P, et al. Systematic review of factors influencing the adoption of information and communication technologies by healthcare professionals. J Med Syst. 2012;36:241–77. 10.1007/s10916-010-9473-4. 20703721 10.1007/s10916-010-9473-4 PMC4011799

[56] Poon EG, Jha AK, Christino M, Honour MM, Fernandopulle R, Middleton B, et al. Assessing the level of healthcare information technology adoption in the United States: a snapshot. BMC Med Inf Decis Mak. 2006;6:1. 10.1186/1472-6947-6-1. 10.1186/1472-6947-6-1 PMC1343543 16396679

[57] Lincoln YS, Guba EE. Research, evaluation, and policy analysis: heuristics for disciplined inquiry. Rev Policy Res. 1986;5:546–65. 10.1111/j.1541-1338.1986.tb00429.x.

[58] Skivington K, Matthews L, Simpson SA, Craig P, Baird J, Blazeby JM et al. A new framework for developing and evaluating complex interventions: update of Medical Research Council guidance. BMJ. 2021;374:n 2061. 10.1136/bmj.n2061. 10.1136/bmj.n2061 PMC8482308 34593508

[59] World Medical Association. Declaration of Helsinki medical research involving human participants. 2024. https://www.wma.net/what-we-do/medical-ethics/declaration-of-helsinki/. Accessed 18 Sept 2025. 10.1001/jama.2024.21972 39425955

