# Supplementary material for: Exploring conditions for implementing daily monitoring of Behavioral and Psychological Symptoms of Dementia (BPSD) in dementia care: insights from a participatory design study
Source: BMC Geriatr. 2026 Mar 11;26:457. doi: 10.1186/s12877-026-07321-0 (PMC13045123; doi:10.1186/s12877-026-07321-0)
Supplement: Supplementary file 1 — Supplementary Material 1. [file 12877_2026_7321_MOESM1_ESM.docx]

# Supplemental Material

**Appendix I – Preparatory Work: Interview Guide for personal assistants, care unit managers, and activity pedagogues**

**Method and Purpose**
Semi-structured interviews will be used to explore how MISU (added information for appendix: name of the previously developed tool) has functioned within personal assistance, group homes, and daily activity services. The findings will inform the main study, in which managers and staff working in dementia care will be interviewed. The ambition is to identify both success factors and challenges related to the use of MISU.

**Background Information**
Gender, professional title, education, years in current profession, years at current workplace

**Introductory Questions**

- How long have you been using MISU in your organization?
- How was the introduction of MISU managed in your unit?

**Implementation/Introduction**

- Can you describe how MISU was implemented at your workplace?
  - Planning?
  - Training?
  - What aspects have worked well?
  - What aspects have worked less well?
  - What would you do differently, or not repeat?

**Use**

- Can you describe how MISU is currently used at your workplace?
- What has the use of MISU contributed to?
  - Which factors have influenced its use and outcomes?
  - What has worked well?
  - What has worked less well?
- Are there any differences between using MISU and your previous approaches to individual-based follow-up?
  - If so, what differences?
- How do you think MISU could be applied in dementia care?
  - What advice would you give in that case?

**Closing**

- Is there anything you would like to add or comment on that we have not yet covered?

**Appendix II – Main Study: Interview Guide for assistant nurses, first-line care managers, registered nurses, members of dementia support teams, researchers, relatives, and occupational therapists**

| **Discussion Area** | **Questions** |
| --- | --- |
| BPSD Registry | • OPENING QUESTION: What is your experience with the BPSD registry? How did you address it most recently? What do you think is most important to consider to prevent/reduce BPSD?  • What skills or relationships are required to identify and reduce/alleviate BPSD early? How do you act and follow up on the actions taken, and what effect does this have?  • What are the most prominent challenges in identifying and assessing BPSD? |
| Measurements in Specific Housing | • OPENING QUESTION: What type of measurements are currently in use outside of the BPSD registry?  • How are measurements initiated?  • Who is responsible for ensuring that certain measurements are conducted? Contact persons? Are these people trained?  • Are there elements missing that would make it easier to work on gathering data in daily work? |
| Conditions for Digital Documentation | • OPENING QUESTION: What systems are currently available for documentation? What access is there to documentation?  • What systems are currently used for documentation of assessments and evaluations?  • How can the documentation be better adapted to your needs?  • Are there differences among staff members in personal ability to document? |
| Variables: What is relevant to follow up regarding BPSD, and how do you see it? | • OPENING QUESTION: Which variables would be relevant to include when working with BPSD? Why?  • What important variables would be most useful for both staff and for long-term patient outcomes?  • Are there any challenges with existing variables that hinder smooth implementation? |
| Consensus and Reliability | • OPENING QUESTION: Are assessments of BPSD reliable? Do you feel confident that other staff could make the same assessments as you do? Why or why not?  • How do you ensure that evaluations remain reliable across different teams or shifts? |
| Routines and Responsibilities | • OPENING QUESTION: What routines are in place for evaluations and documentation?  • Are there specific routines for determining who is responsible for documentation and when it is done?  • Do these routines need improvement to better fit within workflows? |
| Use of Results | • OPENING QUESTION: How are results from evaluations used?  • Are the results shared in team discussions, or used for patient care improvement?  • Do you find the feedback loop from BPSD evaluations sufficient or lacking? |
| Potential Value and Challenges | • OPENING QUESTION: What potential value do you see in implementing tools like Daily-BPSD?  • Are there specific challenges you foresee with implementing these tools? |
| Preparation for Meeting 2 | • OPENING QUESTION: What do you feel is most important to continue discussing in Meeting 2?  • Any other thoughts or questions? |
